# Supplementary material for: Pervasive Somatic Mutations in NF1 Drive Cell Type and Spatially Specific Clonal Selection in Neurofibromatosis
Source: bioRxiv. 2026 Sep 30:2026.09.29.755447. Preprint. [Version 1] doi: 10.64898/2026.09.29.755447 (PMC13648584; doi:10.64898/2026.09.29.755447)
Supplement: Supplementary file 1 [file NIHPP2026.09.29.755447V1-supplement-1.pdf]

bioRxiv preprint doi: https://doi.org/10.64898/2026.09.29.755447; this version posted September 30, 2026. The copyright holder for this preprint (which was not certified by peer review) is the author/funder, who has granted bioRxiv a license to display the preprint in perpetuity. It is made available under a CC-BY 4.0 International license.

Supplementary Materials

Supplementary Tables

Table S1. (Supplemental_Tables.xlsx)
Type Cohort demographics and germline *NF1* mutation status.

Table S2. (Supplemental_Tables.xlsx)
Targeted sites in bulk targeted DNA sequencing panel.

Table S3. (Supplemental_Tables.xlsx)
Somatic *NF1* point mutations identified by bulk targeted DNA sequencing.

Table S4. (Supplemental_Tables.xlsx)
Mosaic chromosomal alterations identified by bulk DNA microarray.

Table S5. (Supplemental_Tables.xlsx)
Targeted sites in snDNA-seq panel.

Table S6. (Supplemental_Tables.xlsx)
Target genes in MERFISH panel.

Supplementary Data

Data S1. (Data_S1_NFvsCTRL_DEG.xlsx)
Differentially expressed genes in snRNA-seq comparing NF-1 donors to control donors.

Data S2. (Data_S2_LOHvsNEU_DEG.xlsx)
Differentially expressed genes in snRNA-seq comparing cells with LOH to cells without LOH.

Supplementary Figures

bioRxiv preprint doi: https://doi.org/10.64898/2026.09.29.755447; this version posted September 30, 2026. The copyright holder for this preprint (which was not certified by peer review) is the author/funder, who has granted bioRxiv a license to display the preprint in perpetuity. It is made available under a CC-BY 4.0 International license.

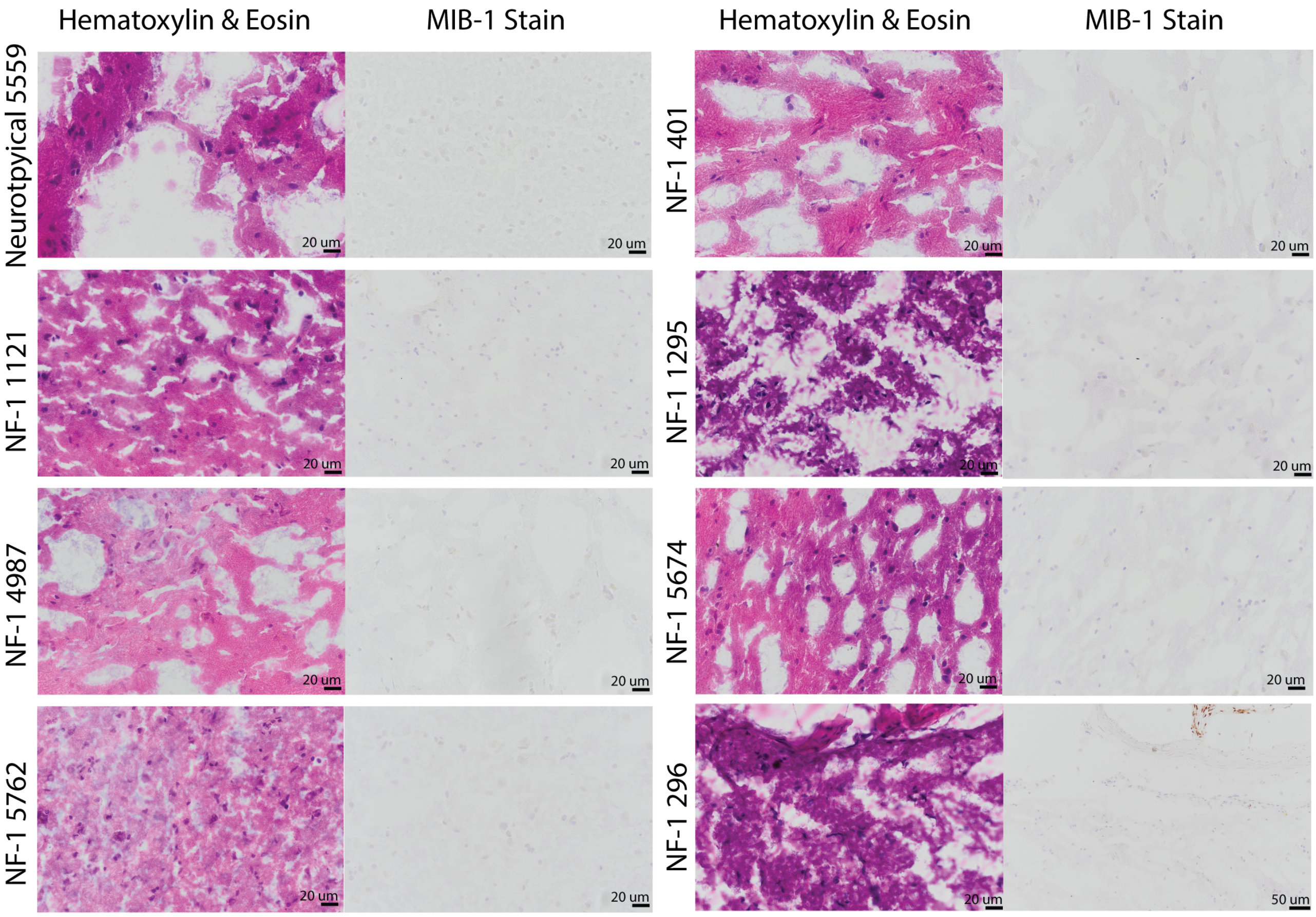

Figure S1: **Histopathological analysis of NF-1 brain sections.** Hematoxylin and eosin (H&E) and Ki-67/MIB-1 (proliferation marker) stains do not show evidence of hypercellularity or increased proliferation characteristic of brain tumors. Histology was performed on available frozen brain tissue.

bioRxiv preprint doi: https://doi.org/10.64898/2026.09.29.755447; this version posted September 30, 2026. The copyright holder for this preprint (which was not certified by peer review) is the author/funder, who has granted bioRxiv a license to display the preprint in perpetuity. It is made available under a CC-BY 4.0 International license.

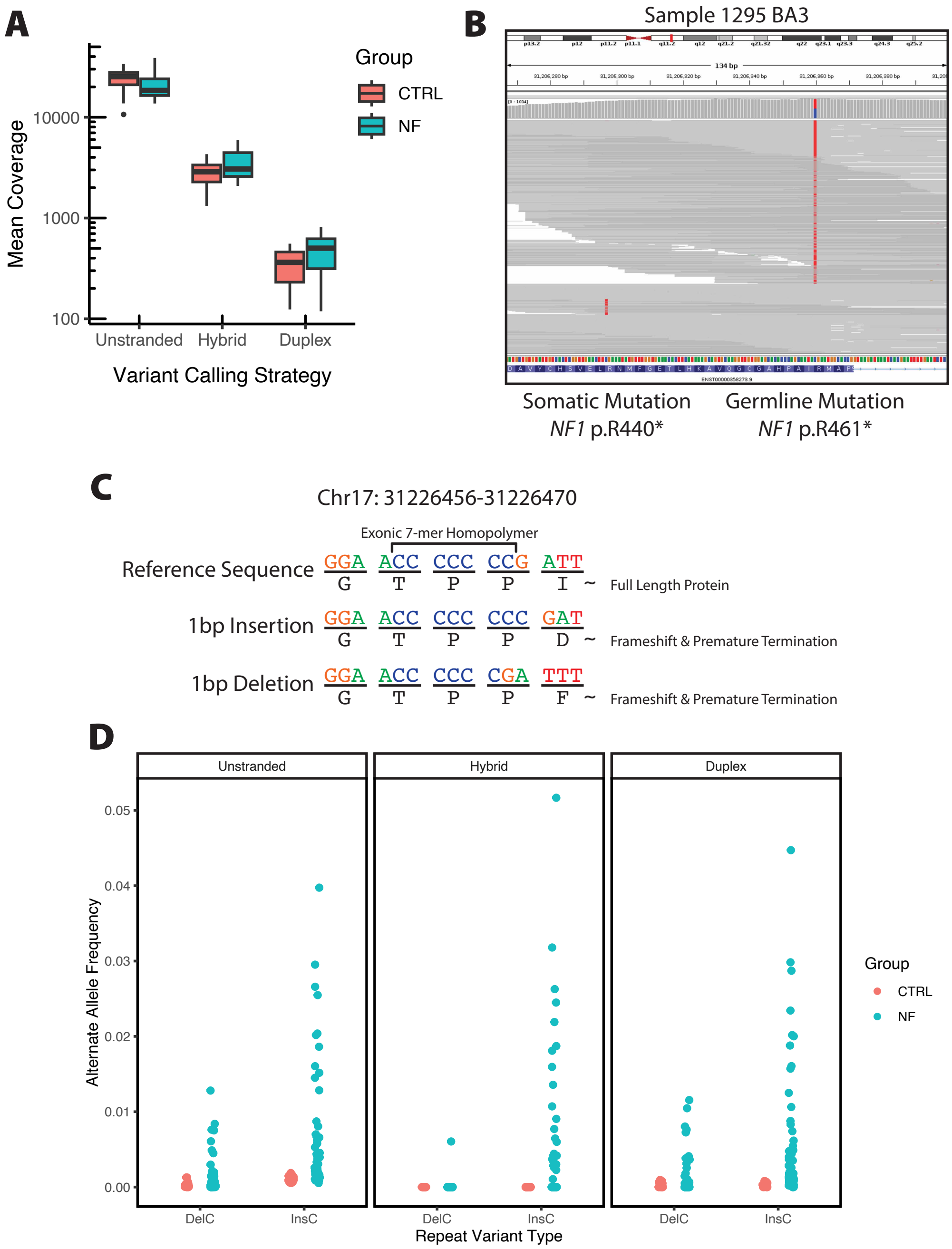

Figure S2: **Targeted *NF1* sequencing metrics and presence of recurrent homopolymer mutations.** (**A**) Mean coverage in targeted regions for each calling strategy: ignoring strand information (unstranded), using information from both molecules with duplex consensus or consensus from a single strand (hybrid), or strictly considering molecules with duplex consensus (duplex). (**B**) Screenshot from IGV showing a somatic and germline mutation identified in BA3 from 1295. Reads spanning both positions show that the somatic and germline variants are in trans orientation. (**C**) Schematic showing a 7-mer homopolymer that is recurrently mutated in samples from individuals with NF-1. (**D**) Allele frequency for single bp insertions or deletions in the 7-mer homopolymer across calling strategies and disease status.

bioRxiv preprint doi: https://doi.org/10.64898/2026.09.29.755447; this version posted September 30, 2026. The copyright holder for this preprint (which was not certified by peer review) is the author/funder, who has granted bioRxiv a license to display the preprint in perpetuity. It is made available under a CC-BY 4.0 International license.

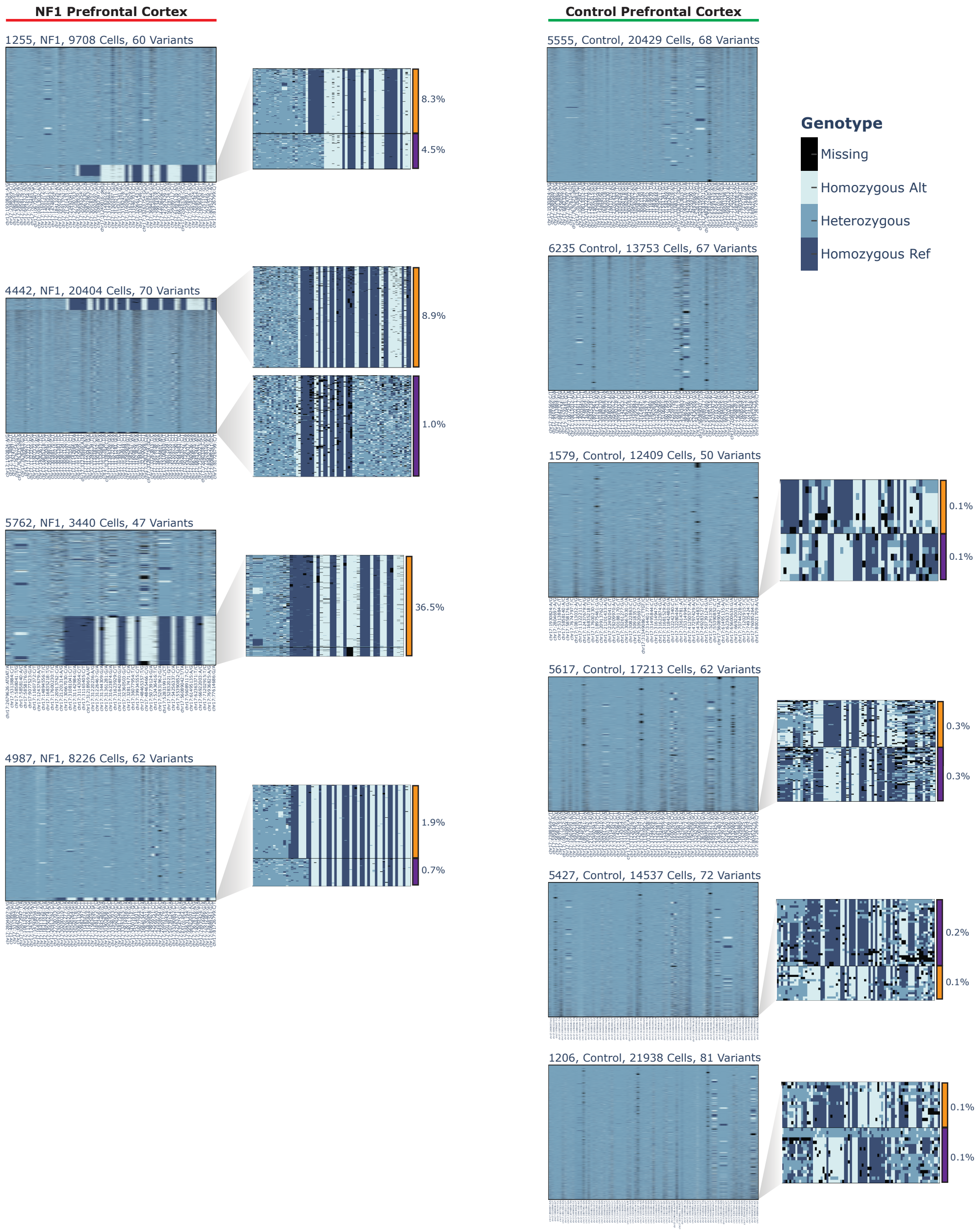

Figure S3: **LOH identified in snDNA-seq.** Cell (row) x variant (column) matrices are shown for 10 prefrontal cortex (BA9) samples (4 NF-1, 6 control) used for snDNA-seq. Each x axis shows germline heterozygous polymorphic markers on chromosome 17 ordered by genomic coordinate. Each row is a single cell assayed. The fill of each tile in the matrix is the called genotype of that cell; either heterozygous (blue), homozygous for the reference allele (dark blue), homozygous for the alternate allele (light blue), or no genotype was called (black). Eight of the samples also have insets showing a zoomed region of a portion of the parent matrix that show either called LOH events (for NF-1 samples) or possible low level LOH that could not be confidently called (in controls). Note that in the NF-1 samples, the LOH events always impact the same allele (visible by pattern of light/dark blue stripes), whereas the possible LOH in controls is present on both alleles in roughly equal frequency (noted by the inverted stripe pattern between the orange and purple cell populations.

bioRxiv preprint doi: https://doi.org/10.64898/2026.09.29.755447; this version posted September 30, 2026. The copyright holder for this preprint (which was not certified by peer review) is the author/funder, who has granted bioRxiv a license to display the preprint in perpetuity. It is made available under a CC-BY 4.0 International license.

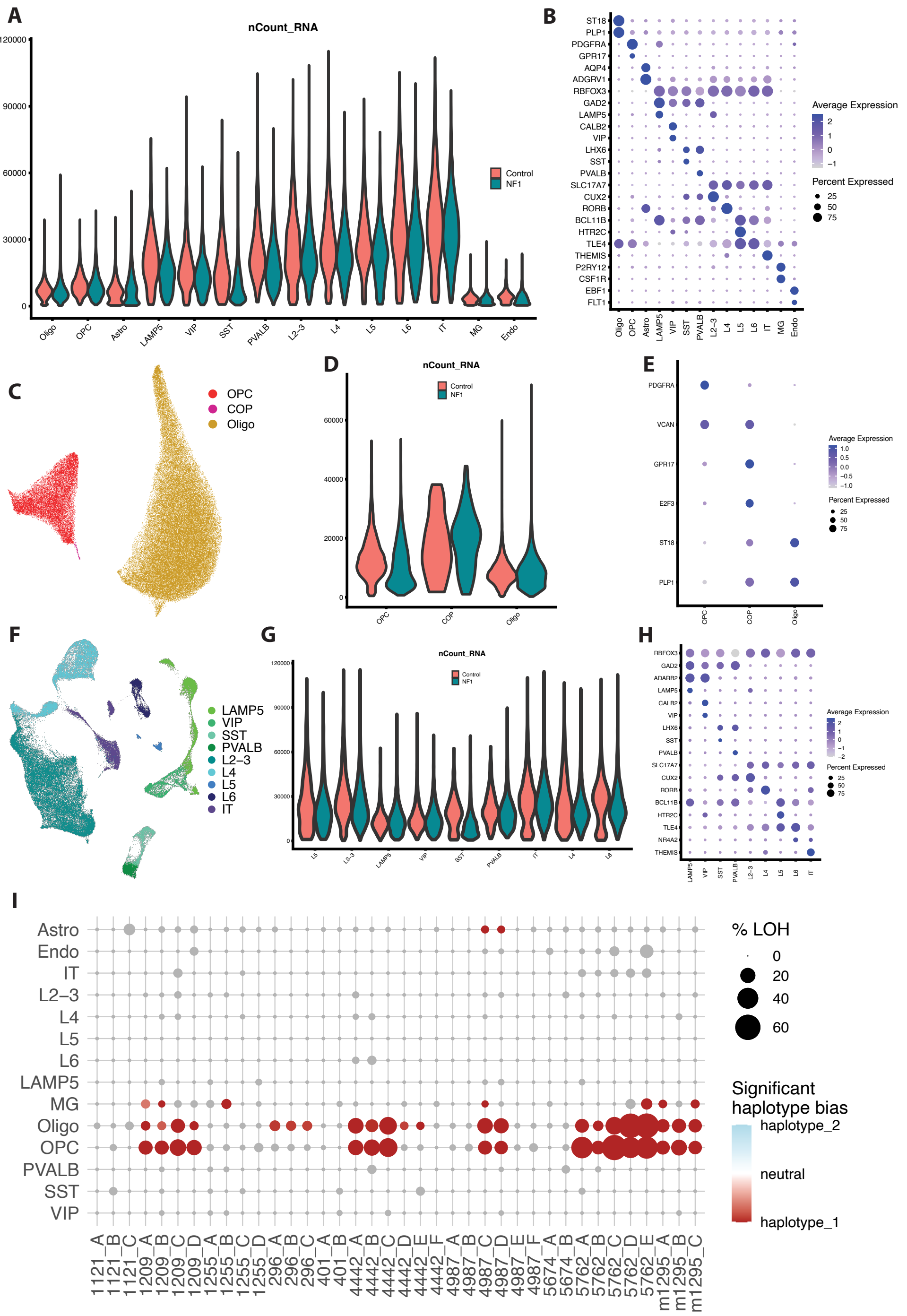

Figure S4: **High-quality snRNA-seq data shows sample-level LOH haplotype bias.** (**A**) Violin plot of UMI count across cell types in unsorted snRNA-seq dataset, split by NF-1 diagnosis. (**B**) Dot plot showing marker gene expression in all cell types in unsorted snRNA-seq dataset. (**C**) UMAP representation of cell types present in SOX10-sorted experiment. Additional Cell Type Abbreviation: COP = Committed Oligodendrocyte Precursors. (**D**) Violin plot of UMI count across cell types in SOX10 sorted snRNA-seq dataset, split by NF-1 diagnosis. (**E**) Dot plot showing marker gene expression in all cell types in SOX10 sorted snRNA-seq dataset. (**F**) UMAP representation of cell types present in NeuN-sorted experiment. (**G**) Violin plot of UMI count across cell types in NeuN sorted snRNA-seq dataset, split by NF-1 diagnosis. (**H**) Dot plot showing marker gene expression in all cell types in NeuN sorted snRNA-seq dataset. (**I**) Haplotype bias for cells called as harboring LOH split by replicates and cell types. Colored dots denote replicates and cell types with significant haplotype bias (FDR < 0.05, binomial test with Benjamini-Hochberg FDR adjustment).

bioRxiv preprint doi: https://doi.org/10.64898/2026.09.29.755447; this version posted September 30, 2026. The copyright holder for this preprint (which was not certified by peer review) is the author/funder, who has granted bioRxiv a license to display the preprint in perpetuity. It is made available under a CC-BY 4.0 International license.

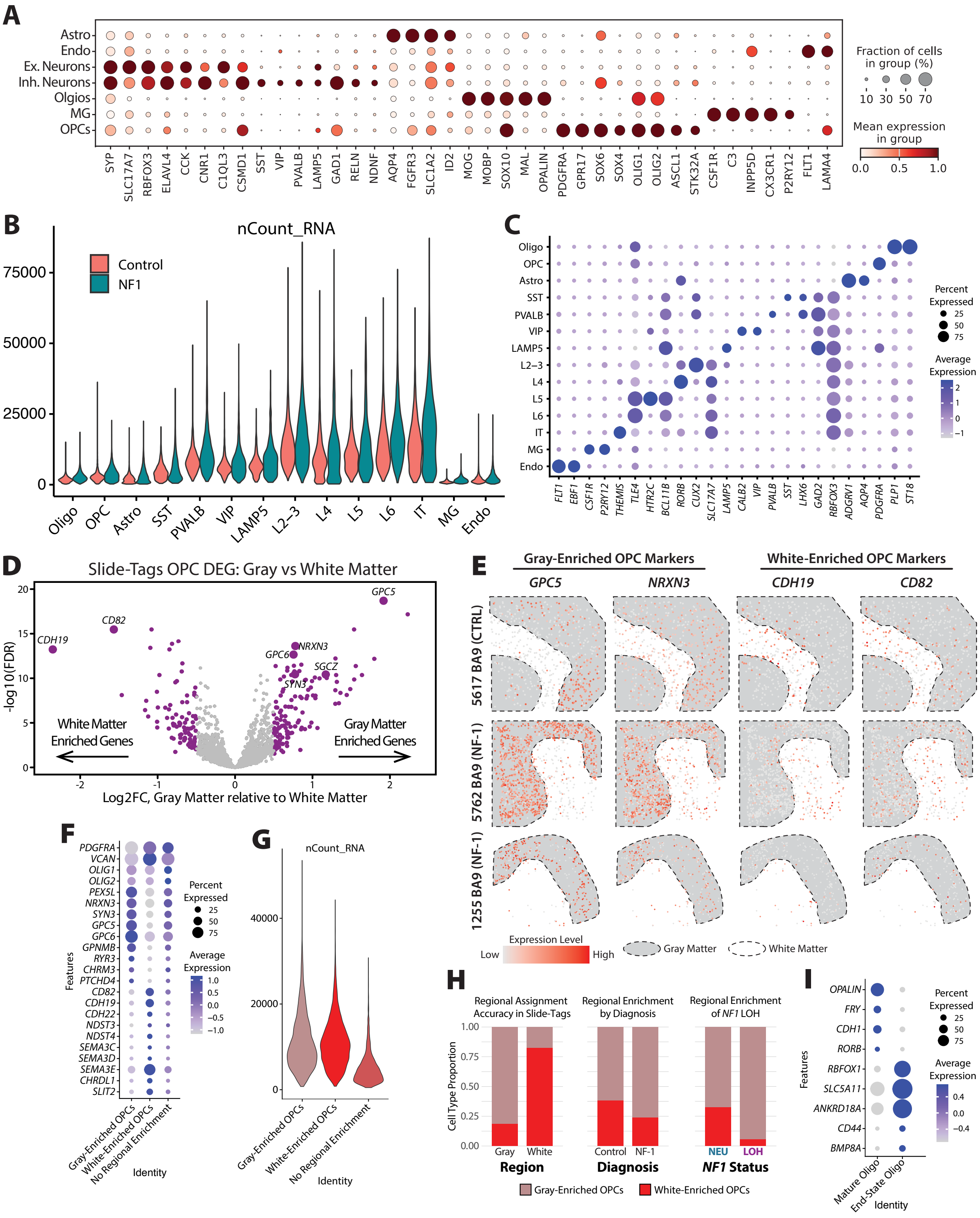

Figure S5: **Spatial transcriptome datasets sequencing and clustering quality metrics.** (**A**) Dot plot showing marker gene expression in all cell types in MERFISH dataset. (**B**) Violin plot of UMI count across cell types in Slide-Tags dataset, split by NF-1 diagnosis. (**C**) Dot plot showing marker gene expression in all cell types in Slide-Tags dataset. (**D**) Volcano plot of region-specific DEGs in OPCs from spatial snRNA-seq data. (**E**) Expression of representative gray/white matter-enriched OPC marker genes. (**F**) Dot plot showing expression of region-specific OPC marker genes across subtypes. (**G**) Violin plot of nCount RNA across gray/white-enriched OPCs showing that the cells with no clear regional enrichment have lower number of transcripts captured. (**H**) Stacked bar plots showing the proportion of transcriptionally determined gray/white-enriched OPCs in (left) gray and white matter in spatial snRNA-seq data, (middle) control and NF-1 donors, and (right) cells with chr17 LOH or neutral. (**I**) Expression of marker genes for OL subtypes.

bioRxiv preprint doi: https://doi.org/10.64898/2026.09.29.755447; this version posted September 30, 2026. The copyright holder for this preprint (which was not certified by peer review) is the author/funder, who has granted bioRxiv a license to display the preprint in perpetuity. It is made available under a CC-BY 4.0 International license.

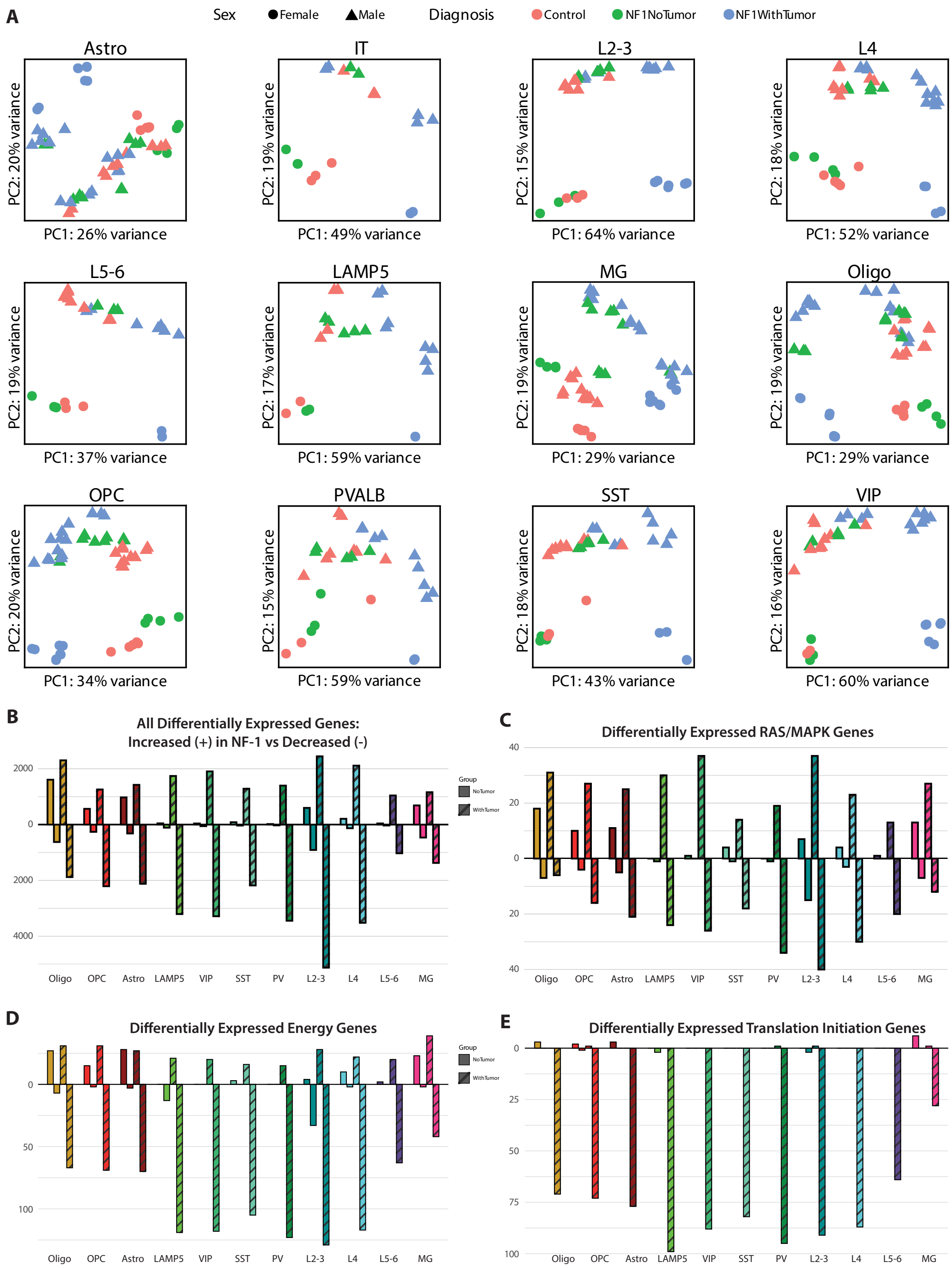

Figure S6: **Principal component clustering of NF-1 donors harboring a brain tumor and corresponding transcriptional differences.** (**A**) Principal component analysis shows all samples across all cell types cluster by sex as expected, but additionally, NF-1 samples from individuals with brain tumors cluster in distinct patterns. PCA generated from DESeq2 normalized counts from each cell type group. Cell Type Abbreviations: Oligo=Oligodendrocytes, OPC=Oligodendrocyte Progenitor Cells, Astro = Astrocytes, LAMP5 = Lysosomal Associated Membrane Protein Family Member 5 Interneurons, VIP= Vasoactive Intestinal Peptide Interneurons, SST = Somatostatin Interneurons, PV = Parvalbumin Basket Cells, L2-3 = Cortical Layer 2/3 Excitatory Neurons, L4 = Cortical Layer 4 Excitatory Neurons, L5-6 = Cortical Layer 5/6 Excitatory Neurons, IT = Intratelencephalic Excitatory Neurons, MG = Microglia. (**B**) Number of differentially expressed (FDR < 0.05 & $|\log_2(FC)| > 0.5$) genes, as well as gene sets from the Reactome database plotted including (**C**) RAS/MAPK, (**D**) energy (glycolysis and oxidative phosphorylation) genes, and (**E**) translation initiation genes, revealing that NF-1 brain samples from individuals with brain tumors show multiple distinct gene expression programs.

bioRxiv preprint doi: https://doi.org/10.64898/2026.09.29.755447; this version posted September 30, 2026. The copyright holder for this preprint (which was not certified by peer review) is the author/funder, who has granted bioRxiv a license to display the preprint in perpetuity. It is made available under a CC-BY 4.0 International license.

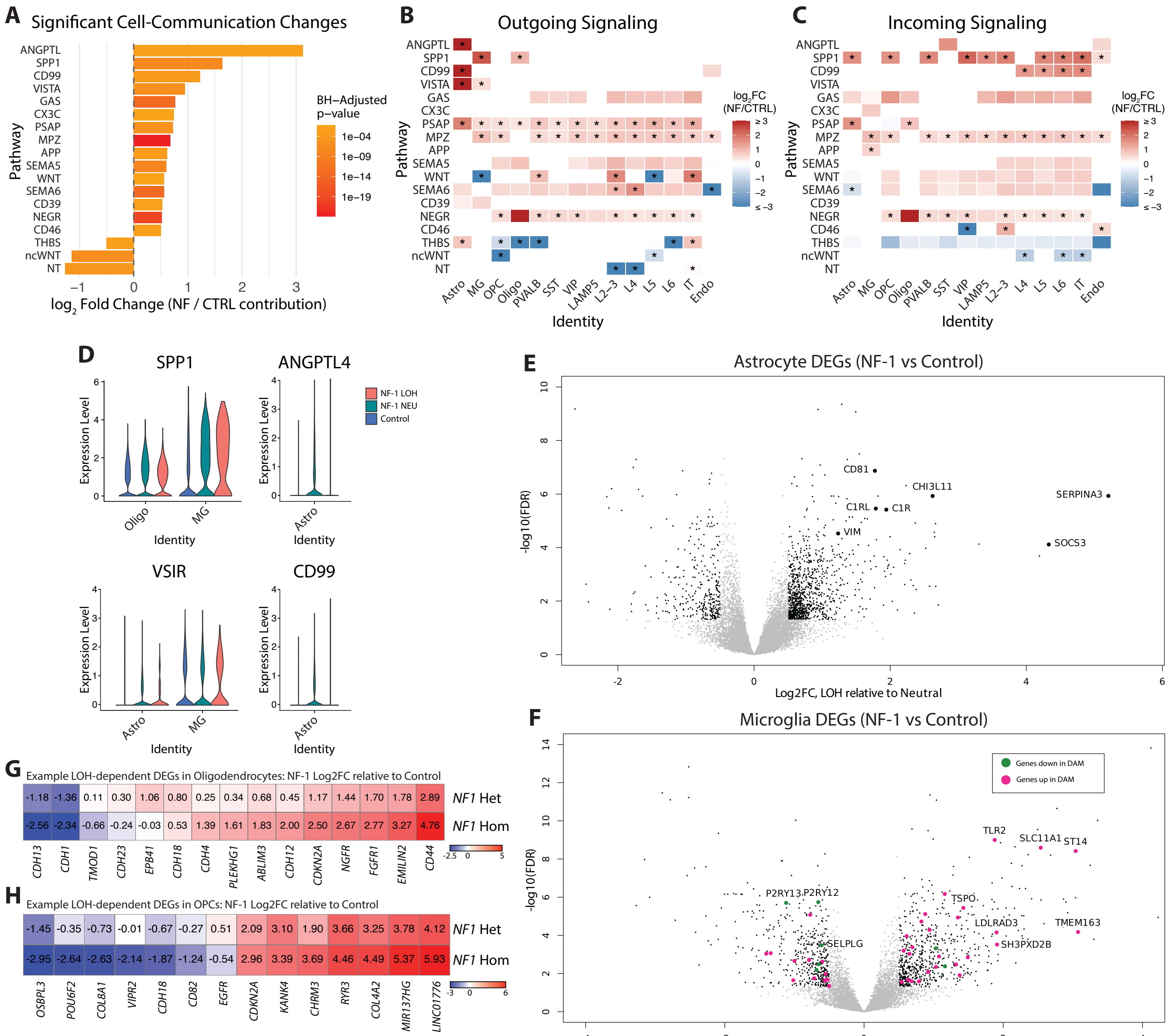

Figure S7: **Cell-cell communication and DEG analysis highlight increased astrocyte reactivity and microglia activation in NF-1.** (**A**) Pathway-level enrichment showing overall shifts in cell-cell communication in NF-1 donors vs controls. (**B**) Heatmap showing pathway enrichment for outgoing signaling (ligand-producing cells) and (**C**) incoming signaling (receptor-producing cells) (* $p < 0.05$ by random permutation testing, Benjamin-Hochberg adjusted). (**D**) Violin plots showing ligand receptor expression from the top four differential pathways in A. (**E**) Volcano plots displaying DGE results in NF-1 without brain tumor astrocytes vs neurotypical OLs, revealing increased reactive astrocyte genes in NF-1. (**F**) Volcano plots displaying DGE results in NF-1 without brain tumor microglia vs neurotypical microglia, revealing increased Disease Associated Microglia (DAM)-like gene expression patterns in NF-1, DAM genes from Keren-Shaul et al. (**G**) Heatmap of significant DEGs in LOH vs Neutral OL DGE demonstrating greater changes in *NF1* LOH OLs than *NF1* Neutral OLs relative to neurotypical OLs. (**H**) Heatmap of significant DEGs in LOH vs Neutral OPC DGE demonstrating greater changes in *NF1* LOH OPCs than *NF1* Neutral OPCs relative to neurotypical OPCs.

bioRxiv preprint doi: https://doi.org/10.64898/2026.09.29.755447; this version posted September 30, 2026. The copyright holder for this preprint (which was not certified by peer review) is the author/funder, who has granted bioRxiv a license to display the preprint in perpetuity. It is made available under a CC-BY 4.0 International license.

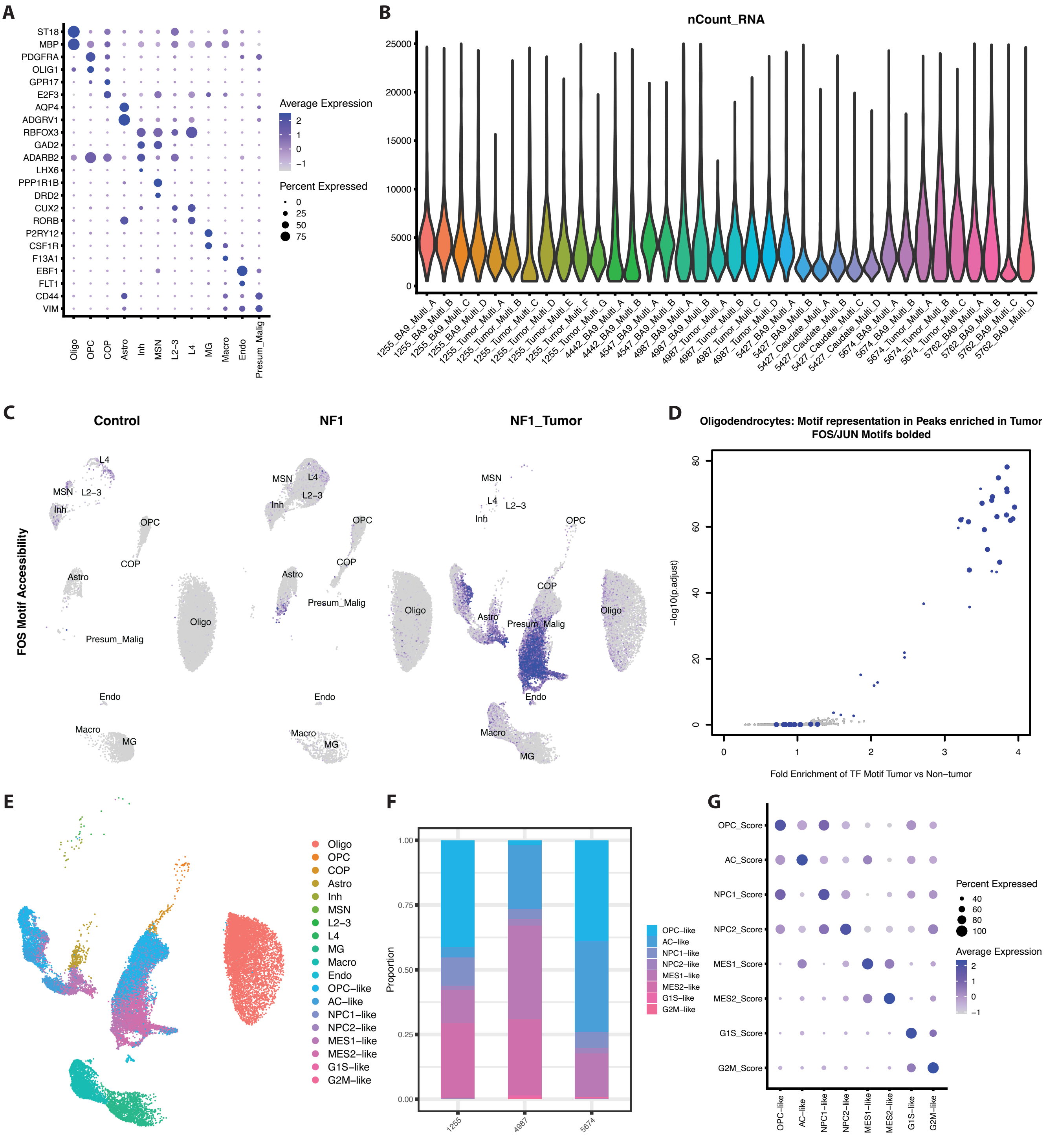

Figure S8: **Marker gene expression, cell state distribution, UMI count, and FOS/JUN motif accessibility in multiome dataset.** (**A**) Dot plot showing marker gene expression in all cell types in multiome dataset. Cell Type Abbreviations: Oligo=Oligodendrocytes, OPC=Oligodendrocyte Progenitor Cells, COP = Committed Oligodendrocyte Progenitors, Astro = Astrocytes, Inh = Inhibitory Neurons, MSN = Medium Spiny Neurons, L2-3 = Cortical Layer 2/3 Excitatory Neurons, L4 = Cortical Layer 4 Excitatory Neurons, MG = Microglia, Macro = Macrophages, Endo = Endothelial Cells, Presum_Malig = Presumed Malignant Cells. (**B**) Violin plot of UMI count across cell types in multiome dataset. (**C**) Feature plot split by sample type showing FOS Motif accessibility greatly increased in presumed malignant nuclei but subtly increased in tumor OLs. (**D**) Enrichment of transcription factor motifs in ATAC peaks more accessible in tumor samples than matched non-tumor samples in OLs; FOS and JUN motifs strongly enriched in tumor-enriched ATAC peaks in OLs. (**E**) UMAP representation of tumor samples, including cell state for presumed malignant nuclei. (**F**) Stacked bar chart showing the distribution of cell states in presumed malignant nuclei across the 3 tumors. (**G**) Dot plot showing expression of cell state modules across malignant cell states.

bioRxiv preprint doi: https://doi.org/10.64898/2026.09.29.755447; this version posted September 30, 2026. The copyright holder for this preprint (which was not certified by peer review) is the author/funder, who has granted bioRxiv a license to display the preprint in perpetuity. It is made available under a CC-BY 4.0 International license.

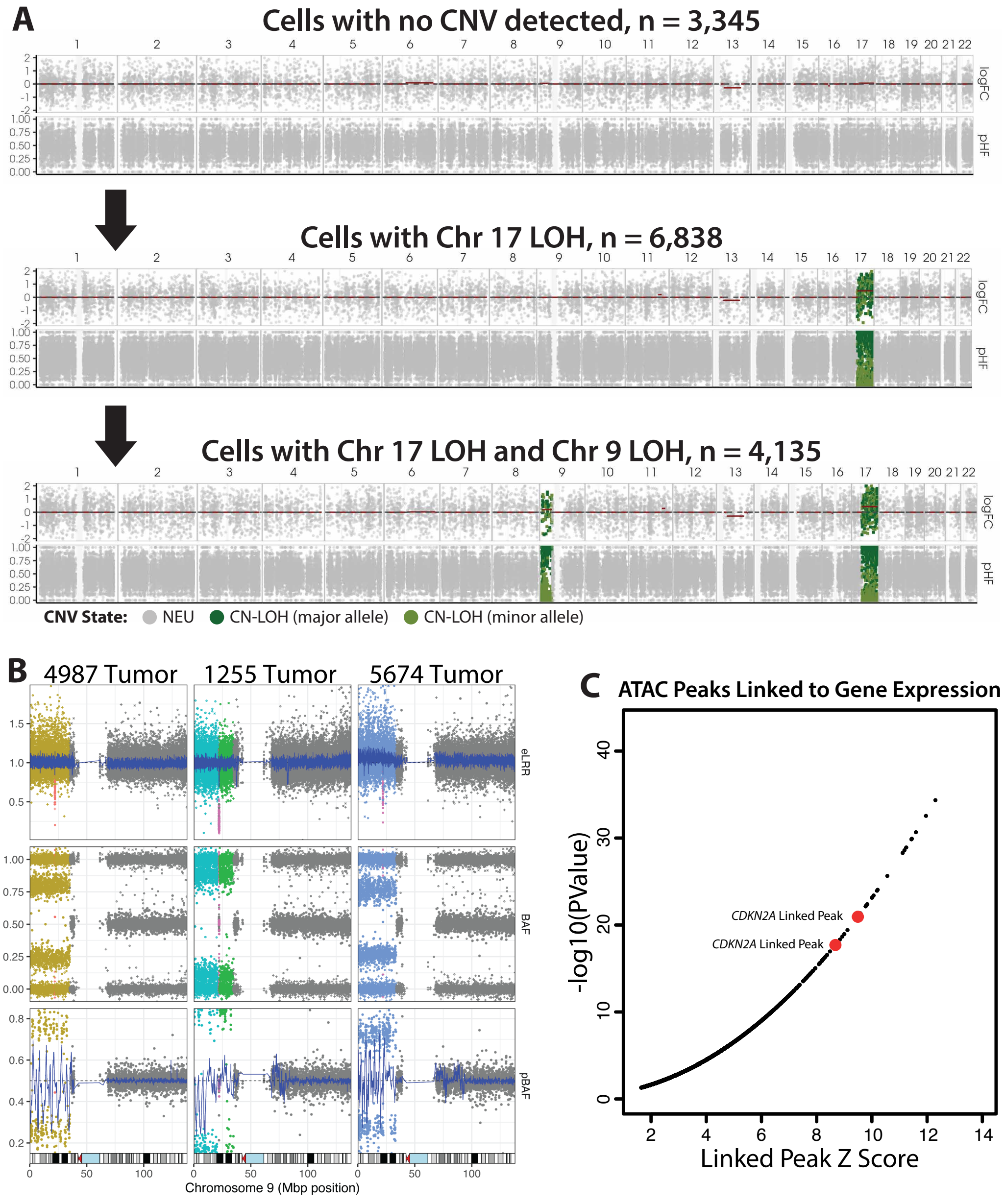

Figure S9: ***CDKN2A* loss occurs after Chromosome 17 LOH and leads to chromosome 9 loss of heterozygosity in all 3 NF-1 tumors.** (**A**) Pseudobulk of snRNA-seq data for three clones identified by Numbat in the tumor from donor 1255. No mCA were identified in the top clone. The middle clone has a single mCA on chromosome 17 which is also present in the bottom clone in addition to an mCA on chromosome 9 overlapping *CDKN2A*. (**B**) MoChA plots showing *CDKN2A* loss in all three NF-associated tumors. The top 3 plots show genotyping intensity and show a drop in intensity corresponding to the location of *CDKN2A* indicating a deletion. In addition to the focal deletion in *CDKN2A* there is also a CN-LOH event encompassing the majority of chr9p including *CDKN2A* which drives the deletion to homozygosity. (**C**) All chromatin peaks significantly linked ($p < 0.05$) to changes in gene expression, with two peaks in *CDKN2A* gene in the top 30.
